# Words of Cheer

**Published:** 1861-12

**Authors:** 


**WORDS OF CHEER.**—It is a grand, good thing in times like these, not only to know that the JOURNAL is not losing money, but that it is actually doing good, making converts to common-sense and a rational mode of living. A stranger writes: "I am a reader of the JOURNAL OF HEALTH. I don't think I ever saw so much common-sense wrapped up in a few pages. Your work on 'HEALTH AND DISEASE' is an excellent one, but your book on 'SLEEP' caps the whole. What next? In my sleeping apartment I have adjusted a cord and two pulleys to the top sash, so that I can lower or raise it at pleasure; at the bottom one I have a piece of wood with notches cut in, so that I can raise or lower it in like manner, as occasion may require. I write not to flatter, but to let you know that there is one soul enjoying to its fullest capacity what thousands of human beings are daily depriving themselves of, namely, a pure atmosphere. May the instruction from the pages of this your book on 'SLEEP,' make many a faint pulse beat with a more healthful and vigorous vitality, and the sickly visage just budding now and waning, may blossom as the rose."

While speaking of our book on "SLEEP," sent post paid for $1.37, we take occasion to commend it anew to those of our subscribers who have not purchased it. To those who can not spare the price, we say, send four new subscribers to the JOURNAL OF HEALTH, and we will send the book free of charge. Its first object is to show, by well-authenticated facts, the important influence had over the health of any one, in spending one third of the whole existence, as is done in sleeping, in a pure atmosphere. We next show how this may be safely secured. The various sources of impurity in our sleeping apartments are pointed out; as also the baleful effects of crowding in sleeping; the certain ill results of the young sleeping with the old; the sick with the well; also the social relations of sleep, as to children sleeping with children; the habits sometimes induced; the results of those habits in after-life; the consequences of those habits; the very certain, safe, and costless mode of correcting them; the importance of control; of securing sound, uninterrupted sleep; how mothers may nurse and train their infants and small children, so as not to interrupt the sleep at night; and the important benefits arising to the children, to the mother, and to the father, in securing these results. There is much in the book of absorbing interest to all cultivated minds, whether young or old. A due attention to its general suggestions would very greatly add to the health, the happiness, the life and purity of any community.

